# Tubulointerstitial Infiltration of M2 Macrophages in Henoch-Schönlein Purpura Nephritis Indicates the Presence of Glomerular Crescents and Bad Clinical Parameters

**DOI:** 10.1155/2019/8579619

**Published:** 2019-01-20

**Authors:** Jisup Kim, Sung-Eun Choi, Keum Hwa Lee, Hyeon Joo Jeong, Jae Il Shin, Beom Jin Lim

**Affiliations:** ^1^Department of Pathology, Yonsei University College of Medicine, Seoul, Republic of Korea; ^2^Department of Pediatrics, Yonsei University College of Medicine, Seoul, Republic of Korea; ^3^Institute of Kidney Disease Research, Yonsei University College of Medicine, Seoul, Republic of Korea

## Abstract

Henoch-Schönlein purpura (HSP) is the most common systemic vasculitis in children, and renal involvement (HSP nephritis, HSPN) is a severe manifestation. HSPN is histologically classified by the International Study of Kidney Disease in Children (ISKDC) based on mesangial hypercellularity and the extent of glomerular crescents. Macrophages, categorized as M1 or M2, frequently infiltrate in various glomerular and tubulointerstitial diseases and infiltration of specific subtypes is associated with disease progression. Therefore, to identify whether infiltration of M1 or M2 macrophages has clinical significance, we quantified the subtypes of macrophages in 49 HSPN specimens and correlated the counts with histologic features and clinical parameters. Higher tubulointerstitial M2 counts were associated with chronic renal failure (CRF), ISKDC classes III-IV, and crescents (*P*<0.001, 0.002, 0.001). Glomerular M2 counts were significantly related to ISKDC classes III-IV and crescents (area under curve, AUC 0.804, 0.833). Tubulointerstitial M2 counts were associated with CRF, ISKDC classes III-IV, and crescents (AUC 0.872, 0.778, 0.830). Tubulointerstitial M2 counts also revealed higher AUC than tubulointerstitial M1 counts for CRF (*P*=0.036) and ISKDC classes III-IV (*P*=0.047). Glomerular M2 counts revealed higher AUC than glomerular M1 counts for ISKDC classes III–IV (*P*=0.024). Tubulointerstitial M2 counts were the most powerful parameter for CRF (AUC 0.872) and revealed even higher AUC than ISKDC classification (AUC 0.716) with borderline significance (*P*=0.086) for CRF. In summary, tubulointerstitial M2 counts were a superior parameter to tubulointerstitial M1 counts and even to ISKDC classification indicating the presence of CRF.

## 1. Introduction

Henoch-Schönlein purpura (HSP) is the most common systemic vasculitis in children. The histologic feature of HSP is leukocytoclastic vasculitis of small vessels, and renal involvement (HSP nephritis, HSPN) is a significant prognostic indicator [1, 2]. The histology and pathogenesis of HSPN are similar to IgA nephropathy and include IgA1-containing immune complex deposits [3]. Although histologically similar, there are different classifications for IgA nephropathy and HSPN. The Oxford classification is a histologic classification of IgA nephropathy, which focuses not only on histologic features, but also on their interobserver reliability and clinical implications [4, 5]. It classifies cases according to the absence or presence of four histologic parameters: mesangial hypercellularity, endocapillary proliferation, segmental sclerosis or adhesion, and tubular atrophy and interstitial fibrosis. For HSPN, the International Study of Kidney Disease in Children (ISKDC) is currently the most widely used classification system. Mesangial hypercellularity and the extent of glomerular crescents are parameters of ISKDC; however, the latter was not considered in the original Oxford classification of IgA nephropathy [4, 5] and has only very recently been included in the revised version [6].

Macrophage infiltration is frequently observed in glomerular and tubulointerstitial diseases. There are several reports that the degree of macrophage infiltration, especially infiltration of specific subtype, is related to the severity of glomerular injury [7] and the progression of tubulointerstitial fibrosis [8]. Activated macrophages are subdivided according to their differentiation as M1 (classically activated) or M2 (alternatively activated) macrophages. M1 macrophages are activated by interferon-*γ* and exert proinflammatory properties, whereas M2 macrophages are activated by interleukin-4 and interleukin-10 and exert anti-inflammatory, immunosuppressive, and extracellular matrix remodeling activities [9]. Glomerular injury and tubular injury cause chemoattraction of macrophages and subsequent inflammatory reactions and tissue remodeling through the production of cytokines, such as transforming growth factor-*β*. A few studies have investigated the significance of macrophage subtype in IgA nephropathy. Ikezumi et al. reported that M2 macrophages were observed in glomeruli and interstitium of early-onset IgA nephropathy, and these cells were correlated with glomerular matrix expansion [10]. Kawasaki et al. observed that severe IgA nephropathy cases, which are resistant to treatment, revealed more frequent M1 macrophage infiltration [11]. In contrast, Li et al. reported that CD163-positive M2 macrophages were responsible for crescent formation and acute tubular injury in IgA nephropathy [12].

It has been recently reported that the endocapillary proliferation and tubular atrophy/interstitial fibrosis aspects of the Oxford classification, together with the crescent formation of the ISKDC classification, are significantly related to the renal survival of HSPN patients [13]. These three features are known to be associated with macrophage infiltration [12, 14–16]. Therefore, we evaluated the macrophage subclasses in renal biopsy specimens of HSPN patients. The quantity of M1 and M2 macrophages were analyzed in relation to histologic features and clinical parameters.

## 2. Materials and Methods

We retrieved 49 cases of biopsy-proven HSPN from the renal biopsy registry and electronic medical records of Severance Hospital, Seoul, Korea. Clinical history and laboratory data were collected by reviewing electronic records. Glomerular filtration rate (GFR) was estimated using the Modification of Diet in Renal Disease study equation [17]. Chronic renal failure (CRF) was defined as a GFR of less than 60 ml/minute/1.73 m^2^ for more than 3 months, which corresponds to chronic kidney disease stage 3-5 according to the National Kidney Foundations Practice Guidelines [18]. Elevation of creatinine (ECr) was defined as elevated serum creatinine greater than or equal to 1.0 mg/dl at the time of biopsy.

Paraffin-embedded renal tissues were cut into 3 *μ*m sections and were stained using the Bond-III automated staining platform (Leica Microsystems, Ltd., Wetzlar, Germany). Antibodies used were anti-human MRP 8/14 (Calprotectin) monoclonal antibody (dilution 1:200; clone 27E10; BMA Biomedicals, Augst, Switzerland) and anti-human CD163 monoclonal antibody (dilution 1:150; clone 10D6; Leica Biosystems, Wetzlar, Germany). After deparaffinization and rehydration by graded alcohol and water, heat-induced antigen retrieval was performed in the Bond Epitope Retrieval Solution 1/2 (Leica Microsystems, Ltd.) at 98°C for 20 minutes.

The number of immunoreactive cells was counted in glomeruli and tubulointerstitium, separately. The average number of positive cells per glomerulus was counted in all glomeruli. Glomeruli showing global sclerosis or crescent formation were excluded from the count. Tubulointerstitial macrophage infiltration was expressed as the average number of positive cells per high power field (HPF, 400x) after counting 10 consecutive HPFs. The number of M1 (MRP 8/14-positive) and M2 macrophages (CD163-positive) was analyzed in relation to histologic and clinical features, such as crescent formation, serum creatinine level, hematuria, and proteinuria. This study was approved by the Institutional Review Board of Gangnam Severance Hospital (3-2015-0067). The Institutional Review Board exempted the authors from obtaining informed consent because of the retrospective nature of this study.

## 3. Statistical Analysis

Statistical comparisons were conducted using an independent Student's t-test for continuous variables and chi-square or Fisher's exact test for categorical variables. The statistical analyses were performed with SPSS version 23.0 (IBM, Armonk, NY, USA). Area under curve (AUC) was compared from receiver operating characteristic curves with 95% confidence intervals (95% CI), and AUC comparisons were performed with MedCalc version 18 (MedCalc Software bvba, Ostend, Belgium) using the DeLong method [19]. Statistical significance was given to results with* P*-values<0.05 or AUC>0.7, and borderline significance was given to* P*-values between 0.05 and 0.10.

## 4. Results

**Figure 1:**
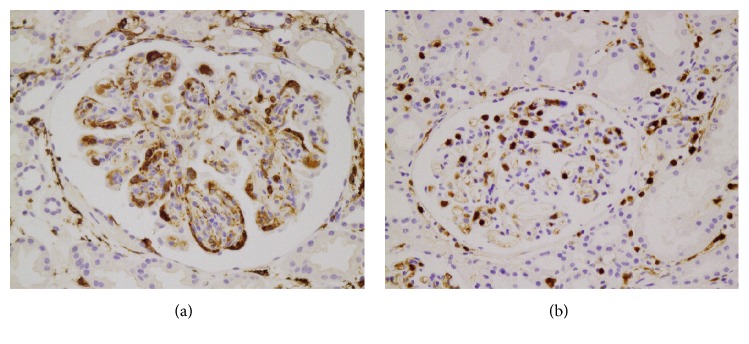
Representative microscopic images of MRP 8/14-positive M1 (a) and CD163-positive M2 macrophage (b) in glomeruli and tubulointerstitium (x400).

The baseline characteristics of 49 HSPN patients, including clinical presentation and laboratory findings, are shown in Table 1. Infiltration of M1 and M2 macrophages was observed in glomeruli and tubulointerstitium (Figures 1(a) and 1(b)). The mean glomerular M1 and M2 counts (± standard deviation, SD) were 1.0±2.8 and 5.7±11.4, respectively, and the mean tubulointerstitial M1 and M2 counts were 0.6±1.8 and 52.6±38.5, respectively. The differences in clinical, laboratory, light microscopic, and immunofluorescence findings between groups with and without ECr at the time of biopsy, CRF at the time of biopsy, and ISKDC classes are described in Tables 1 and 2. ECr cases were associated with older age (*P*<0.001), higher body mass index (kg/m^2^,* P*=0.003), lower GFR (*P*<0.001), and reduced serum albumin (*P*=0.003) (Table 1). CRF cases were associated with older age (*P*<0.001), higher serum creatinine (*P*=0.008), and reduced serum albumin (*P*=0.006) (Table 1). ISKDC class III-IV cases were associated with older age (*P*=0.047) and hypoalbuminemia (*P*=0.049) (Table 1). ECr cases were associated with increased presence (*P*=0.047) and glomerular involvement of crescents (*P*=0.036) (Table 2). CRF cases were associated with presence of crescents (*P*=0.008), increased glomerular crescent involvement (*P*=0.037), and IgM deposits (*P*=0.024) (Table 2). ISKDC class III-IV cases were associated with increased glomerular crescent involvement (*P*<0.001) and presence of crescents (*P*<0.001) (Table 2).

The quantities of M1 and M2 macrophages were compared according to the presence of ECr, CRF, ISKDC classes III-IV, or presence of crescents. Both glomerular and tubulointerstitial M1 counts tended to be higher in cases with ECr (*P*=0.088, 0.055) (Table 3). Tubulointerstitial M2 counts were higher in cases with ECr (*P*=0.001), CRF (*P*<0.001), ISKDC classes III-IV (*P*=0.002), and presence of crescents (*P*=0.001) (Table 3). There was no difference in the number of M1 and M2 macrophages in glomeruli or tubulointerstitium according to the presence of endocapillary proliferation (data not shown).

The AUC was obtained to evaluate the correlation to ECr, CRF, ISKDC classes III-IV, and presence of crescents. Glomerular M1 counts were significantly associated with ECr (AUC 0.724) in contrast to glomerular M2 counts, which were not associated with ECr (AUC 0.617) (Table 4). Glomerular M2 counts were significantly associated with ISKDC classes III-IV (AUC 0.804) and presence of crescents (AUC 0.833) (Table 4). M1 counts in tubulointerstitium and likewise M1 counts in glomeruli were associated with ECr (AUC 0.769) but not associated with CRF, ISKDC classes III-IV, or presence of crescents (Table 4). Tubulointerstitial M2 counts were associated with ECr (AUC 0.818), CRF (AUC 0.872), ISKDC classes III-IV (AUC 0.778), and presence of crescents (AUC 0.830) (Table 4). ISKDC classification was significantly associated with ECr (AUC 0.711) and CRF (AUC 0.716) (Table 4).

M1 counts in glomeruli, as well as M1 and M2 counts in tubulointerstitium, revealed higher AUC than ISKDC classification for the association with ECr but without statistical significance (*P*=0.693, 0.399, 0.212). Additionally, the AUC difference between tubulointerstitial M1 (AUC 0.769) and M2 counts (AUC 0.818) was not statistically significant (*P*=0.618).

**Figure 2:**
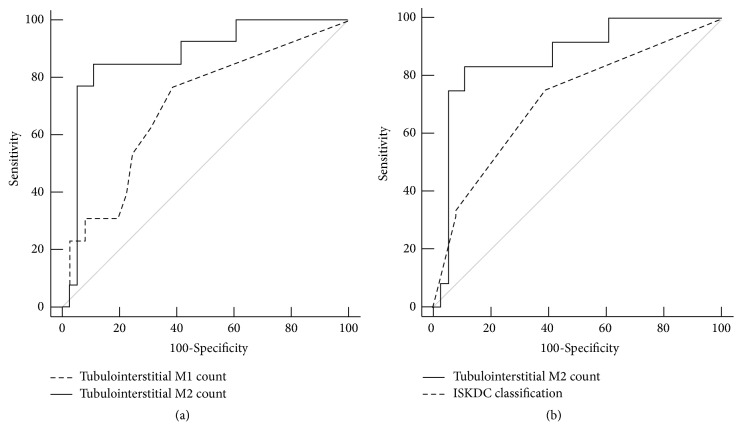
**Area under curve (AUC) comparison of receiver operating characteristic (ROC) curves of tubulointerstitial M1 and M2 macrophages counts (a) and tubulointerstitial M2 counts and ISKDC classification (b) for the association with chronic renal failure**. (a) AUC of tubulointerstitial M2 counts [0.872 (95% CI 0.745 to 0.950)] was wider than tubulointerstitial M1 counts [0.700 (95% CI 0.552 to 0.822)] (*P*=0.036). (b) AUC of tubulointerstitial M2 counts [0.866 (95% CI 0.736 to 0.947)] was wider than AUC of ISKDC [0.716 (95% CI 0.568 to 0.837)] (*P*=0.086). The AUC 95%, CI, and* P*-values in AUC comparison were obtained from DeLong method. ISKDC, International Study of Kidney Disease in Children.

**Figure 3:**
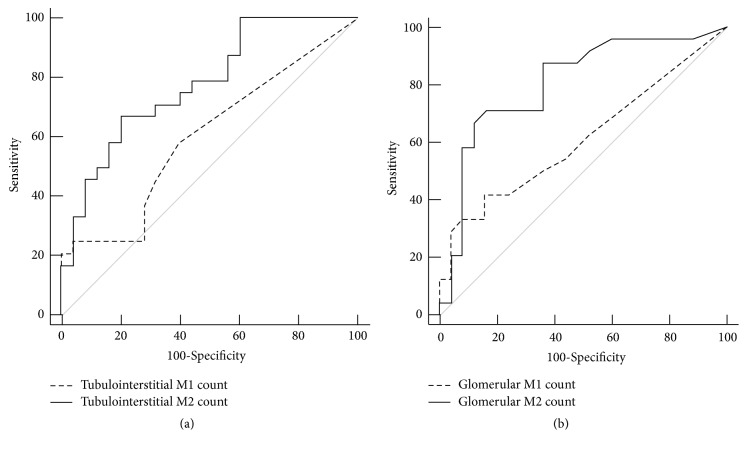
**Area under curve (AUC) comparison of receiver operating characteristic (ROC) curves of tubulointerstitial M1 and M2 macrophage counts (a) and glomerular M1 and M2 macrophage counts (b) for the association with ISKDC classes III-IV**. (a) AUC of tubulointerstitial M2 counts [0.778 (95% CI 0.637 to 0.884)] was wider than AUC of tubulointerstitial M1 counts [0.602 (95% CI 0.452 to 0.739)] (*P*=0.047). (b) AUC of glomerular M2 counts [0.804 (95% CI 0.666 to 0.904)] was wider than AUC of glomerular M1 counts [0.611 (95% CI 0.461 to 0.747)] (*P*=0.024). The AUC, 95% CI, and* P*-values in AUC comparison were obtained from DeLong method. ISKDC, International Study of Kidney Disease in Children; CI, confidence interval.

Conversely, tubulointerstitial M2 counts revealed higher AUC than tubulointerstitial M1 counts for the association with CRF (*P*=0.036) and ISKDC classes III-IV (*P*=0.047) (Table 4 and Figures 2(a) and 3(a)). Glomerular M2 counts revealed higher AUC than glomerular M1 counts for the association with ISKDC classes III-IV (*P*=0.024) (Table 4 and Figure 3(b)). Tubulointerstitial M2 counts were the most powerfully associated factor with CRF (AUC 0.872) and revealed even higher AUC than ISKDC classification with borderline significance (*P*=0.086) for CRF (Table 4 and Figure 2(b)).

## 5. Discussion

Glomerular and tubulointerstitial macrophage infiltration plays a significant role in the progression of various renal diseases. Renal glomerular and tubulointerstitial fibrosis, a crucial final common pathway that dictates renal function and survival, is tightly spatiotemporally related to macrophage infiltration [20]. Macrophages produce supporting factors for myofibroblasts, such as galectin-3, transforming growth factor-*β*, insulin-like growth factor 1, and platelet-derived growth factor, and also play a role in the deposition and organization of the extracellular matrix by modulating the balance of metalloproteinases and tissue inhibitors of metalloproteinases [20, 21]. Moreover, degradative metalloproteinases released by macrophages can damage the tubular basement membrane leading to epithelial-to-mesenchymal transition [21]. Just as epithelial-to-mesenchymal transition is an important origin of bone-marrow-derived myofibroblasts, the macrophage itself is also an important origin of myofibroblasts through the macrophage-myofibroblast transition [20, 22–24].

There have been several studies showing that the dominance of M1 or M2 macrophages leads to progression of kidney diseases. M1 polarization has been suggested to be pathogenic in antiglomerular basement membrane glomerulonephritis (demonstrated by interferon-*γ*-augmented adoptive transfer) [25, 26], early stage ischemia-reperfusion injury (reflecting damage after renal allograft) [27], and Adriamycin nephropathy (demonstrated by adoptive transfer of CpG DNA-activated M1 macrophages) [28]. In contrast, M2 macrophages have been suggested to be pathogenic in the later stage of ischemia-reperfusion injury [27]. Studies have demonstrated M1 polarization is associated with diabetic nephropathy in streptozotocin-induced type I diabetic mouse and rat models [26, 29, 30]. However, another study revealed M2 polarization is associated with diabetic nephropathy in rats with hyperalbuminuric streptozotocin-induced type I diabetes [26, 31]. Some studies demonstrated M2 polarization is associated with exacerbation of lupus nephritis [32, 33], whereas another study showed M1 macrophages are associated with the onset of lupus nephritis [34].

There are a few studies showing that M1 and M2 macrophages take part in IgA nephropathy and HSPN [10–12]. More specifically, M2 macrophages are related to crescent formation in IgA nephropathy and HSPN [7, 12]. In this study, we analyzed the significance of M1 and M2 macrophages in HSPN in association with clinical parameters. M1 and M2 macrophages were counted in glomeruli and tubulointerstitium, separately. Of note, glomerular infiltration was counted only in nonsclerotic glomeruli without crescents, because it is already known that the number of M2 macrophages is significantly correlated with crescents [12] and glomerulosclerosis [35].

Our analysis demonstrated that the quantity of glomerular M2 macrophages showed significant association with the development of crescents and higher ISKDC classes, of which the main discriminating factor is the number of crescents. Therefore, high numbers of glomerular M2 macrophages correlated with the development of crescents, which is consistent with previous studies [12]. In contrast, glomerular M1 macrophage counts were higher in the ECr group than the non-ECr group with borderline significance, whereas the association with ECr by AUC was significant. This correlation might be partially explained by the fact that M1 macrophages have proinflammatory properties, and M2 macrophages have profibrotic properties [36, 37].

Interestingly, we found that the quantity of tubulointerstitial M2 macrophages was more prominently associated with the development of ECr, CRF, crescent formation, and higher ISKDC classes in both absolute counts and AUC. Increased glomerular injury could have contributed to more significant tubulointerstitial inflammation. Alternatively, elevated tubulointerstitial inflammation and fibrosis could have affected the progression of glomerular injury [38]. As HSPN is primarily a disease of the glomeruli, the former is more plausible. However, it has been recently reported that preexisting tubulointerstitial injury is a deteriorating factor for the development and progression of glomerular injury [39]. Therefore, there is a possibility that tubulointerstitial M2 macrophages induced more severe glomerular injury with crescent formation. Our observation that M2 macrophages had more significant clinical translation is supported by previous studies. In an* in vitro* study, M2, but not M1 macrophages promoted epithelial-to-mesenchymal transition in cisplatin-induced nephrotoxicity, which was characterized by apoptosis of tubular epithelial cells through inflammatory mediators and oxidative stress [40]. In an ischemia/reperfusion injury mouse model, M2, but not M1 macrophages revealed an important role in the progression of fibrosis during acute kidney injury-to-chronic kidney disease transition [41].

ISKDC classification considers only mesangial hypercellularity and the extent of glomerular crescents, but other significant components, such as vessels and tubulointerstitium, are omitted [3, 42, 43]. Additionally, ISKDC classification has been challenged by contradictory clinical outcomes in several reports [44–47]. A new semiquantitative classification, including activity index and chronicity index, has been suggested recently on the grounds of improved sensitivity for clinical outcomes [48]. We identified the superiority of tubulointerstitial M2 macrophage counts to the current ISKDC classification by AUC comparison analysis. As the DeLong test is a conservative statistical method [49], the borderline significance of the superiority of tubulointerstitial M2 counts to ISKDC classification in terms of CRF shown in this study may provide meaningful clinical implication. In contrast to ISKDC classification, which concentrates only on histologic features, tubulointerstitial M2 counts could guide potential targeted therapy as it plays a key role in the dynamic process of renal fibrogenesis [50, 51].

As HSPN is frequently diagnosed in pediatric patients in very small biopsy specimens, this study has additional clinical implications. When a biopsy of HSPN is small and no crescent is found, immunohistochemistry for M2 macrophages would be beneficial. Although this study does not provide a cut-off value, elevated glomerular M2 counts may suggest unsampled crescents, and higher tubulointerstitial M2 counts may indicate a poor prognosis regarding acute and chronic renal function deterioration.

There are several limitations in this study. First, the outcomes, such as ECr and CRF, are based on initial clinical data at the time of biopsy. Second, survival analysis could not be performed due to lack of follow-up clinical data. Third, possible confounding factors, such as age, body mass index, and treatment modality, could not be adjusted due to the retrospective nature of this study.

In conclusion, tubulointerstitial M2 counts were superior to tubulointerstitial M1 counts and were even superior to ISKDC classification in association with CRF of HSPN patients. Tubulointerstitial M2 counts would not only aid to predict outcomes and guide clinical practice, especially when only a small biopsy sample is available, but may also provide insight for potential therapeutic targets to prevent fibrosis and renal failure. Future studies employing larger cohorts are necessary to establish a grading system for tubulointerstitial M2 counts.

## Figures and Tables

**Table 1:** Clinical and laboratory findings between patient groups based on GFR and ISKDC classification.

**Factors**	**All patients**	**Renal failure**			**Chronic renal failure**			**ISKDC classification**		
	**(n=49)**	**ECr** ^**c**^ ** (n=17)**	**Non-ECr (n=32)**	***P***	**CRF** ^**d**^ ** (n=13)**	**Non-CRF (n=36)**	***P***	**Class I-II (n=25)**	**Class III–IV (n=24)**	***P***
	**Mean±SD/ n (%)**	**Mean±SD/ n (%)**	**Mean±SD/ n (%)**		**Mean±SD/ n (%)**	**Mean±SD/ n (%)**		**Mean±SD/ n (%)**	**Mean±SD/ n (%)**	
**Baseline characteristics**										
Age (years)	39.6±19.8	**54.2±21.3**	31.8±14.0	**<0.001**	**64.2±16.5**	30.6±11.8	**<0.001**	34.0±10.8	**45.4±25.1**	**0.047**
Sex (Male:Female)	(26:23)	(11:6)	(15:17)	0.234	(5:8)	(21:15)	0.218	(14:11)	(12:12)	0.674
BMI (kg/m^2^)	21.7±3.8	**23.9±3.8**	20.5±3.3	**0.003**	23.1±3.3	21.1±3.9	0.107	22.4±4.3	20.9±3.2	0.178
**Clinical presentations**										
Arthralgia	7 (14.3)	2 (11.8)	5 (15.6)	0.999	1 (7.7)	6 (16.7)	0.658	4 (16.0)	3 (12.5)	0.999
Abdominal pain	6 (18.4)	3 (17.6)	6 (18.8)	0.999	1 (7.7)	8 (22.2)	0.412	5 (20.0)	4 (16.7)	0.999
Melena	3 (6.1)	2 (11.8)	1 (3.1)	0.273	1 (7.7)	2 (5.6)	0.999	2 (8.0)	1 (4.2)	0.999
Gross hematuria	16 (32.7)	6 (35.3)	10 (313)	0.774	4 (30.8)	12 (33.3)	0.999	10 (40.0)	6 (25.0)	0.263
**Laboratory findings**										
GFR^a^	90.3±39.5	57.7±34.8	108.2±29.5	**<0.001**	38.8±14.4	109.4±26.4		80.9±45.0	99.6±31.4	0.104
Creatinine (mg/dl)	1.0±0.7	1.6±1.0	0.7±0.2		**1.7±1.1**	0.8±0.2	**0.008**	1.2±1.0	0.8±0.3	0.095
Albumin (mg/dl)	4.1±1.0	3.6±1.0	**4.4±0.9**	**0.003**	3.5±0.9	**4.3±0.9**	**0.006**	4.1±1.3	4.2±0.5	0.677
Hypoalbuminemia (Albumin < 3.0 mg/dl)	7 (14.3)	**6 (35.3)**	1 (3.1)	**0.005**	**4 (30.8)**	3 (8.3)	0.070	1 (4.0)	**6 (25.0)**	**0.049**
24hr proteinuria (g/day)	2114.7±2161.6	2,706.1±2,849.3	1,892.1±1,803.0	0.358	2,273.6±2,971.1	2,086.7±1,776.9	0.864	2,751.6±2,525.2	1,494.5±1,521.8	0.115
Nephrotic syndrome range of proteinuria^b^	11 (37.9)	4(44.4)	7 (35.0)	0.694	3 (33.3)	8 (40.0)	0.999	4 (28.6)	7 (46.7)	0.316

^a^GFR was estimated using the Modification of Diet in Renal Disease study equation.

^b^Nephrotic syndrome range of proteinuria is above 2.5 g/day in total urine.

^c^Definition of elevation of creatinine (ECr) is the status of high creatinine ≥ 1.0 mg/dl.

^d^Definition of chronic renal failure (CRF) is the status of low glomerular filtration rate (GFR), which is below 60 ml/min/1.73 m^2^ for more than 3 months.

BMI, body mass index; GFR, glomerular filtration rate; ISKDC, International Study of Kidney Disease in Children.

**Table 2:** Light microscopy and immunofluorescence findings for patient groups based on GFR and ISKDC classification.

**Factors**	**Renal failure**			**Chronic renal failure**			**ISKDC classification**		
	**ECr (n=17)**	**Non-ECr (n=32)**	***P*** ^**a**^	**CRF (n=13)**	**Non-CRF (n=36)**	***P*** ^**a**^	**Class I-II (n=25)**	**Class III–IV (n=24)**	***P*** ^**a**^
	**Mean±SD or n (%)**	**Mean±SD or n (%)**		**Mean±SD or n (%)**	**Mean±SD or n (%)**		**Mean±SD or n (%)**	**Mean±SD or n (%)**	
**Light microscopy studies**									
Number of glomeruli evaluated	13.8±6.6	19.5±9.6	**0.037**	13.1±6.7	19.1±9.3	**0.046**	17.0±8.1	18.1±10.0	0.696
% Glomeruli involved by crescent	**13.6±14.6**	4.7±8.1	**0.036**	**13.6±13.5**	5.7±10.0	**0.037**	0.0±0.0	**15.8±11.8**	**<0.001**
Presence of sclerotic glomeruli [crescent]	**10 (62.5)**	10 (32.3)	**0.047**	**9 (75.0)**	11 (31.4)	**0.008**	0 (0.0)	**20 (87.0)**	**<0.001**
Presence of endocapillary proliferation	2 (11.8)	2 (6.3)	0.602	1 (7.7)	3 (8.3)	0.999	2 (8.0)	2 (8.3)	0.999
**Immunofluorescence studies**									
IgG	0.4±0.7	0.3±0.5	0.564	0.5±0.7	0.3±0.5	0.358	0.4±0.5	0.3±0.6	0.590
IgA	1.8±0.9	1.6±0.8	0.340	1.6±0.9	1.7±0.8	0.614	1.8±0.8	1.5±0.8	0.259
IgM	0.3±0.3	0.2±0.2	0.120	**0.3±0.3**	0.2±0.2	**0.024**	0.2±0.2	0.3±0.3	0.245
C3	0.7±0.7	0.5±0.4	0.358	0.7±0.7	0.6±0.5	0.626	0.6±0.4	0.6±0.6	0.671
C4	0.0±0.0	0.0±0.1	0.083	0.0±0.1	0.0±0.1	0.788	0.0±0.1	0.0±0.1	0.585
C1q	0.0±0.0	0.0±0.1	0.161	0.0±0.0	0.0±0.1	0.396	0.0±0.1	0.0±0.0	0.161
Fibrinogen	1.4±1.0	1.1±0.8	0.211	1.3±0.8	1.2±0.8	0.718	1.1±0.8	1.3±0.9	0.424

^a^Chi-square test, Mantel-Haenszel Chi-square test, or Fischer exact test for categorical variables; *P*-value < 0.05 was considered statistically significant.

GFR, glomerular filtration rate; ISKDC, International Study of Kidney Disease in Children.

**Table 3:** Comparison of M1 and M2 macrophage counts according to patient groups based on GFR and ISKDC classification.

**Factors**	**ECr** ^**a**^	**Non-ECr** ^**a**^	***P*** ^**c**^	**CRF** ^**b**^	**Non-CRF** ^**b**^	***P*** ^**c**^	**Class I-II**	**Class III-IV**	***P*** ^**c**^	**Crescents**	**No crescents**	***P*** ^**c**^
	**(n=17) Mean±SD**	**(n=32) Mean±SD**		**(n=13) Mean±SD**	**(n=36) Mean±SD**		**(n=25) Mean±SD**	**(n=24) Mean±SD**		**(n=20) Mean±SD**	**(n=27) Mean±SD**	
**Glomeruli**												
M1	2.3±4.4	0.3±0.8	0.088	2.5±5.0	0.4±0.9	0.156	0.4±0.9	1.7±3.8	0.115	2.0±4.1	0.4±0.8	0.099
M2	8.7±14.7	4.1±9.1	0.176	9.7±16.6	4.2±8.7	0.274	3.4±10.3	8.0±12.3	0.164	9.2±13.1	3.4±9.9	0.094
**Tubulointerstitium**												
M1	1.5±2.8	0.1±0.2	0.055	0.9±1.5	0.5±1.9	0.468	0.2±0.4	1.0±2.5	0.118	1.2±2.7	0.2±0.3	0.089
M2	**81.1±44.7**	37.4±24.0	**0.001**	**86.4±33.9**	40.4±32.6	**<0.001**	35.8±24.5	**70.1±42.9**	**0.002**	**73.7±41.8**	35.7±23.3	**0.001**

^a^Definition of elevation of creatinine (ECr) is the status of high creatinine ≥1.0 mg/dl.

^b^Definition of chronic renal failure (CRF) is the status of low GFR, which is below 60 ml/min/1.73 m^2^ for more than 3 months.

^c^Independent two-sample t-test for continuous variables; *P*-value <0.05 was considered statistically significant.

GFR, glomerular filtration rate; ISKDC, International Study of Kidney Disease in Children.

**Table 4:** Area under curve (AUC) comparison of receiver operator characteristic (ROC) curves for the association with renal failure or ISKDC classification.

**Factors**	**ECr** ^**a**^		**CRF** ^**b**^		**ISKDC III-IV**		**Crescents**	
	AUC	95% C.I.	AUC	95% C.I.	AUC	95% C.I.	AUC	95% C.I.
Glomerular M1 count	0.724	0.578 to 0.842	0.656	0.507 to 0.786	0.611	0.461 to 0.747	0.637	0.484 to 0.772
Glomerular M2 count	0.617	0.467 to 0.752	0.610	0.460 to 0.746	0.804	0.666 to 0.904	0.833	0.696 to 0.926
Tubulointerstitial M1 count	0.769	0.627 to 0.878	0.700	0.552 to 0.822	0.602	0.452 to 0.739	0.696	0.545 to 0.822
Tubulointerstitial M2 count	0.818	0.682 to 0.914	0.872	0.745 to 0.950	0.778	0.637 to 0.884	0.830	0.692 to 0.923
ISKDC classification	0.711	0.562 to 0.833	0.716	0.568 to 0.837				

^a^Definition of elevation of creatinine (ECr) is the status of high creatinine ≥1.0 mg/dl.

^b^Definition of chronic renal failure (CRF) is the status of low GFR, which is below 60 ml/min/1.73 m^2^ for more than 3 months.

ISKDC, International Study of Kidney Disease in Children.

## Data Availability

The data used to support the findings of this study are included within the article.
